# Studying alternative splicing regulatory networks through partial correlation analysis

**DOI:** 10.1186/gb-2009-10-1-r3

**Published:** 2009-01-09

**Authors:** Liang Chen, Sika Zheng

**Affiliations:** 1 Molecular and Computational Biology, Department of Biological Sciences, University of Southern California, Los Angeles, California 90089, USA; 2 Howard Hughes Medical Institute, University of California, Los Angeles, MRL 6-619, Los Angeles, California 90095, USA

## Abstract

**Background:**

Alternative pre-mRNA splicing is an important gene regulation mechanism for expanding proteomic diversity in higher eukaryotes. Each splicing regulator can potentially influence a large group of alternative exons. Meanwhile, each alternative exon is controlled by multiple splicing regulators. The rapid accumulation of high-throughput data provides us with a unique opportunity to study the complicated alternative splicing regulatory network.

**Results:**

We propose the use of partial correlation analysis to identify association links between exons and their upstream regulators or their downstream target genes (exon-gene links) and links between co-spliced exons (exon-exon links). The partial correlation analysis avoids taking the ratio of two noisy random variables, exon expression level and gene expression level, so that it achieves a higher statistical power. We named this analysis procedure pCastNet (partial Correlation analysis of splicing transcriptome Network). Through studies of known alternative exons, conservation patterns, relative positions, functional annotations, and RT-PCR experiments, we concluded that pCastNet can effectively identify exon-gene or exon-exon links. We further found that gene pairs with exon-gene or exon-exon links tend to have similar functions or are present in the same pathways. More interestingly, gene pairs with exon-gene or exon-exon links tend to share *cis*-elements in promoter regions and microRNA binding elements in 3' untranslated regions, which suggests the coupling of co-alternative-splicing, co-transcription-factor-binding, and co-microRNA-binding.

**Conclusions:**

Alternative splicing regulatory networks reconstructed by pCastNet can help us better understand the coordinate and combinatorial nature of alternative splicing regulation. The proposed tool can be readily applied to other high-throughput data such as transcriptome sequencing data.

## Abstract 2

The identification of links between exons and their regulators or targets and between co-spliced exons in human, mouse and rat provides novel insights into the alternative splicing regulatory network.

## Background

Alternative pre-mRNA splicing is an important gene regulation mechanism for expanding proteomic diversity in higher eukaryotes. It has been estimated that 59-74% of human genes are alternatively spliced [1,2], and abnormal mRNA splicing contributes to many human diseases [3-5]. The alternative splicing of multiple pre-mRNAs is tightly regulated and coordinated, which is an essential component for many biological processes, including nervous system development and programmed cell death [6,7]. In the process of alternative splicing, splicing regulators bind to various pre-mRNAs and affect a large number of exons. Meanwhile the splicing pattern of a specific exon is determined by multiple pre-mRNA-binding proteins [8,9]. Therefore, it will be particularly interesting to study how the splicing of a group of exons is co-regulated and how the splicing of an exon is combinatorially controlled by multiple regulators.

With advancements in high-throughput technologies, such as Affymetrix exon arrays, various types of junction arrays, or high-throughput sequencing, it is feasible to study alternative splicing on a genomic scale. Current studies have centered on the differential analysis of alternative splicing. To identify exons with differential splicing, we must account for differential transcription of a gene itself. In Affymetrix exon arrays, both exon-level intensity and gene-level intensity are estimated. Gene-level-normalized exon intensity, which is defined as the ratio of the exon intensity to the gene intensity, has been widely used to remove the transcription effect when studying splicing. A significant difference in the normalized exon intensity (NI) indicates that this exon has different inclusion or exclusion rates between two conditions. For example, in ExACT, developed by Affymetrix [10,11], the NI is calculated as the ratio of the exon intensity to the gene intensity. Then, the 'splicing index' value is calculated by taking the log ratio of the NI in sample 1 to the NI in sample 2 to identify exons alternatively spliced between two samples.

Multiple groups have nicely surveyed the complexities of alternative splicing in various tissues and cell lines and observed tissue-specific alternative splicing events mainly through differential analysis [1,11,12]. These events are valuable for investigating the function of alternative splicing in phenotypic diversity. However, their regulatory interactions remain largely unknown; for example, one can hardly speculate on the relationship or the regulators of two exons co-enriched in a specific tissue. In combination with motif analysis, one can further study motif enrichment in a group of tissue-specific alternative exons [13,14]. However, such analysis is constrained by the limited knowledge of splicing regulators and their *cis-*regulatory motifs. The motifs of some splicing regulators have not yet been identified and some RNA binding proteins have almost identical binding motifs. Except for a few splicing factors (for example, FOX proteins), the degenerative nature of binding motifs of splicing regulators further confounds analysis. Several groups have used microarrays in conjunction with manipulation of splicing regulator expression or crosslinking immunoprecipitation (CLIP) of splicing regulators to identify their indirect or direct targets [15,16]. Such studies provide the most valuable data for dissecting alternative splicing regulation centered on one splicing regulator of interest.

Instead of performing differential analysis, we propose to study alternative splicing regulatory networks based on pair-wise co-expression associations of exons and genes across multiple conditions. This can provide a direct association link between two exons or between one exon and one gene. Such association links can be used to infer regulatory or functional relationships between two nodes. In this study, we have used exon array data for human, mouse, and rat across 11 tissues to study alternative splicing regulatory networks. To study the co-splicing patterns of exons, we can intuitively calculate the NI for every exon across multiple conditions and then calculate the correlation between the NIs of two exons. However, the high-level of noise inherent to exon arrays will make the correlation unstable. Indeed, some studies using the NI approach have reported low validation rates (21-56%) for the identification of alternative splicing events [10,17,18]. The possible reason is that the distribution of the ratio of two random variables is often heavy-tailed if the noise level for the two random variables is high [19]. In other words, if the noise level is high, the ratio between the exon intensity and the gene intensity is not stable and it remains a special statistical challenge to derive appropriate test statistics. For example, we considered a constitutive exon and the gene it belongs to. Exon-level and gene-level intensities were simulated according to a bivariate normal distribution. The correlation between the exon-level intensity and the gene-level intensity was set as 0.9 to satisfy that the exon is a constitutive exon. A total of 1,000 expression levels were simulated. As shown in Additional data file 1, when the noise level is high, the NI can be as small as 0.5 or as high as 3 even if the exon is a constitutive exon.

Instead of using the ratio between the exon intensity and the gene intensity, we can perform correlation studies on the exon intensity directly. To remove the transcription effect in the exon intensity, we propose to apply partial correlation analysis. A partial correlation coefficient is the correlation between two variables, with the effects of other variables removed. For example, in order to exclude the possibility that a high exon-exon (EE) correlation is due to either the gene-level association or the association between one exon and the gene that the other exon belongs to, we calculate the partial correlation coefficients between the two exons conditioning on one or two genes. If the partial correlations are still high, we declare that there is an association between the two exons and this association represents a co-splicing relationship. In addition to EE co-splicing links, we also studied exon-gene (EG) links where the high correlation between an exon and a gene is not due to the gene-gene (GG) association. Partial correlation analysis has been applied to gene co-expression network studies [20-22].

In this study we have used exon array data for human, mouse, and rat across 11 tissues. The proposed methods can be readily applied to RNA-Seq data. We want to point out that the co-splicing relationship can be condition-specific. With the rapid accumulation of high-throughput exon array or RNA-Seq data, we will be able to reconstruct dynamic regulatory networks under different conditions in the near future.

## Results

### Determining gene-gene, exon-gene and exon-exon links using pCastNet

Three types of associations were considered for a pair of gene: GG, EG, and EE associations. Using pCastNet (partial Correlation analysis of splicing transcriptome Network), the Pearson correlation coefficient for GG associations was calculated between gene 1 (g_1_) and gene 2 (g_2_) and denoted as ${\text{r}}_{{\text{g}}_{1}{\text{g}}_{2}}$. For EG associations, considering an exon (e_1_) of gene 1 (g_1_) and gene 2 (g_2_), as well as the Pearson correlation coefficient ${\text{r}}_{{\text{e}}_{1}{\text{g}}_{2}}$, the partial correlation coefficient between e_1 _and g_2 _conditioning on g_1 _was calculated as ${\text{r}}_{{\text{e}}_{1}{\text{g}}_{2}•{\text{g}}_{1}}$. The partial correlation can be interpreted as the association between e_1 _and g_2 _after removing the effect of g_1_. If the partial correlation is high, the association between e_1 _and g_2 _is not due to the correlation between g_1 _and g_2_. Otherwise, e_1 _can be a constitutive exon of g_1 _and the association between e_1 _and g_2 _is due to the correlation between the two genes. For EE associations, the correlation between an exon (e_1_) of gene 1 (g_1_) and an exon (e_2_) of gene 2 (g_2_) was calculated as ${\text{r}}_{{\text{e}}_{1}{\text{e}}_{2}}$. We also calculated the partial correlations ${\text{r}}_{{\text{e}}_{1}{\text{e}}_{2}•{\text{g}}_{1}}$, ${\text{r}}_{{\text{e}}_{1}{\text{e}}_{2}•{\text{g}}_{2}}$ and ${\text{r}}_{{\text{e}}_{1}{\text{e}}_{2}•{\text{g}}_{1}{\text{g}}_{2}}$ to exclude the possibility that the EE correlation is due to the EG or GG correlation. In summary, if the *p*-value for ${\text{r}}_{{\text{g}}_{1}{\text{g}}_{2}}$ is significant, we declared a GG link between gene 1 and gene 2. If the *p*-values for both ${\text{r}}_{{\text{e}}_{1}{\text{g}}_{2}}$ and ${\text{r}}_{{\text{e}}_{1}{\text{g}}_{2}•{\text{g}}_{1}}$ are significant, we declared an EG link between e_1 _and g_2_. This association is not due to GG association. If the *p*-values for ${\text{r}}_{{\text{e}}_{1}{\text{e}}_{2}}$, ${\text{r}}_{{\text{e}}_{1}{\text{e}}_{2}•{\text{g}}_{1}}$, ${\text{r}}_{{\text{e}}_{1}{\text{e}}_{2}•{\text{g}}_{2}}$, and ${\text{r}}_{{\text{e}}_{1}{\text{e}}_{2}•{\text{g}}_{1}{\text{g}}_{2}}$ are significant, we declared an EE link between the two exons e_1 _and e_2_. The association is not due to GG or EG associations.

### Simulation studies on the performance of pCastNet

**Figure 1:**
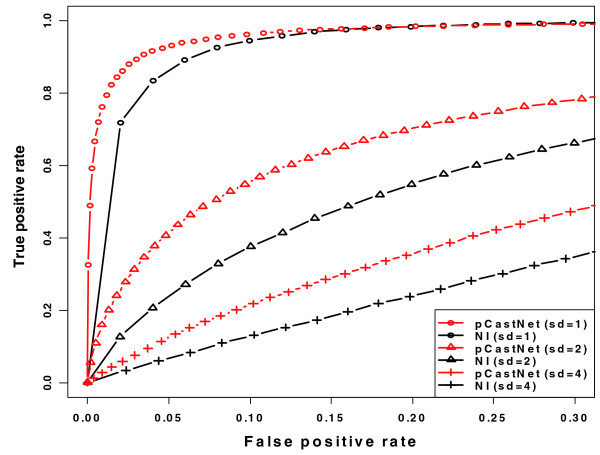
**ROC (receiver operating characteristic) curves of pCastNet and the NI-based approach**. The x-axis is the false positive rate and the y-axis is the true positive rate (power). Red lines are for pCastNet and black lines are for the NI-based approach. The standard deviation of expression level is 1 (circles), 2 (triangles), or 4 (crosses). Simulation procedures can be found in Materials and methods.

We performed simulation studies to illustrate the relative performance of pCastNet. A total of five genes were considered. Each of them has five constitutive exons and one alternative exon. The five alternative exons have the same inclusion rate relative to their gene levels. Thus, the five alternative exons are co-spliced. Exon intensity data were simulated for ten tissues. Gene-level intensity was estimated as the average intensity of the five constitutive exons for each gene. In pCastNet, the correlation and partial correlations between each pair of exons belonging to different genes were calculated as ${\text{r}}_{{\text{e}}_{1}{\text{e}}_{2}}$, ${\text{r}}_{{\text{e}}_{1}{\text{e}}_{2}•{\text{g}}_{1}}$, ${\text{r}}_{{\text{e}}_{1}{\text{e}}_{2}•{\text{g}}_{2}}$, and ${\text{r}}_{{\text{e}}_{1}{\text{e}}_{2}•{\text{g}}_{1}{\text{g}}_{2}}$. In the NI-based approach, the correlation between the NI values of each pair of exons across ten tissues was calculated as r. Different *p*-value thresholds were used to declare whether there is a co-splicing relationship between two exons. Figure 1 shows the ROC (receiver operating characteristic) curves of pCastNet (red) and the NI-based approach (black). Three scenarios were considered: the standard deviation of the exon intensity is 1 (circles), 2 (triangles), or 4 (crosses). pCastNet consistently performed better than the NI-based approach. When the variance of the exon intensity is large (2^2 ^or 4^2^), the power (true positive rate) of pCastNet is almost 50-100% higher than that of the NI-based approach given the same false positive rate. The true positive rates and the false positive rates are the average values across 1,000 simulations for each scenario.

### Choice of significance threshold

The choice of significance threshold remains a major challenge for co-expression network studies. Previous studies have typically relied on a data-independent constant correlation threshold. Zhang and Horvath [23] proposed a weighted gene co-expression network approach. They used soft thresholding instead of hard thresholding to better identify GG links. This method needs a scale-free topology criterion to estimate the involved parameters. Other topology-based approaches include clustering coefficient-based threshold selection developed by Elo *et al *[24]. Because there has been little study on the topology of alternative splicing regulatory networks, we avoided topology-based methods and instead propose a false discovery rate (FDR) approach. Specifically, we used the approach proposed by Efron [25] to control the expected FDR conditioning on a dependence effect parameter A. For GG, EG, and EE networks, hypotheses were performed to test the significance of pair-wise correlations. The dependence among hypotheses is largely ignored in traditional FDR control methods [26,27], despite the fact that correlations among hypotheses may be high for genomics studies [28]. In contrast, the conditional false discovery expectation takes the dependence of hypotheses into account and, therefore, achieves a more accurate estimate of FDR. For GG links, *t*-test statistics (${\text{r}}_{{\text{g}}_{1}{\text{g}}_{2}}\sqrt{\left(\text{n}-2)/(1-{\text{r}}_{{\text{g}}_{1}{\text{g}}_{2}}^{2}\right)}$) were converted to z-values directly. For EG and EE links, *t*-test statistics (${\text{r}}_{{\text{e}}_{1}{\text{g}}_{2}}\sqrt{\left(\text{n}-2)/(1-{\text{r}}_{{\text{e}}_{1}{\text{g}}_{2}}^{2}\right)}$, ${\text{r}}_{{\text{e}}_{1}{\text{g}}_{2}•{\text{g}}_{1}}\sqrt{\left(\text{n}-3)/(1-{\text{r}}_{{\text{e}}_{1}{\text{g}}_{2}•{\text{g}}_{1}}^{2}\right)}$, ${\text{r}}_{{\text{e}}_{1}{\text{e}}_{2}}\sqrt{\left(\text{n}-2)/(1-{\text{r}}_{{\text{e}}_{1}{\text{e}}_{2}}^{2}\right)}$, ${\text{r}}_{{\text{e}}_{1}{\text{e}}_{2}•{\text{g}}_{1}}\sqrt{\left(\text{n}-3)/(1-{\text{r}}_{{\text{e}}_{1}{\text{e}}_{2}•{\text{g}}_{1}}^{2}\right)}$, ${\text{r}}_{{\text{e}}_{1}{\text{e}}_{2}•{\text{g}}_{2}}\sqrt{\left(\text{n}-3)/(1-{\text{r}}_{{\text{e}}_{1}{\text{e}}_{2}•{\text{g}}_{2}}^{2}\right)}$, and ${\text{r}}_{{\text{e}}_{1}{\text{e}}_{2}•{\text{g}}_{1}{\text{g}}_{2}}\sqrt{\left(\text{n}-4)/(1-{\text{r}}_{{\text{e}}_{1}{\text{e}}_{2}•{\text{g}}_{1}{\text{g}}_{2}}^{2}\right)}$) were first converted to z-values. The distribution of the minimum absolute z-value was estimated by a multivariate normal distribution; then, the minimum absolute z-value was further transformed to final z-values. Under the null hypotheses, the final z-values follow the standard normal distribution. By comparing the histogram of z-values and the standard normal distribution, we can estimate the dispersion parameter A that reflects the dependence among hypotheses. Then we can calculate the conditional FDR. However, the number of declared links is very sensitive to the conditional FDR threshold (Table 1). Therefore, instead of applying a threshold on the conditional FDR directly, we estimated the sparseness of a network according to the conditional FDR and then chose a threshold on the sparseness. The sparseness of a network is defined as the percentage of true links among all possible node pairs. The threshold selection has several advantages: first, the corresponding correlation thresholds are data dependent; second, we can derive an accurate estimate of the number of falsely declared links taking into consideration the dependence among hypotheses; and third, we can integrate prior information about the sparseness of networks if this information is available. Here we chose the sparseness threshold as 0.02%; this threshold corresponds to a reasonable conditional FDR and total number of declared GG, EG, and EE links. We also tried thresholds of 0.01% and 0.005%. The results discussed in the remaining of this paper are similar, although the number of links differs significantly (Table 1).

**Table 1:** Sparseness of networks and corresponding conditional FDR, z-value threshold, the number of GG, EG, EE links (n), and the range of correlations and partial correlations

	Human			Mouse			Rat		
			
Sparseness	0.02%	0.01%	0.005%	0.02%	0.01%	0.005%	0.02%	0.01%	0.005%
GG									
cFDR	0.227	0.206	0.185	0.05	0.031	0.02	0.045	0.025	0.015
z	4.42	4.61	4.79	4.77	5.02	5.25	4.8	5.07	5.31
n	13,552	6,523	3,202	12,014	5,878	2,893	2,672	1,307	653
|r_g1g2_|	≥ 0.947	≥ 0.957	≥ 0.965	≥ 0.964	≥ 0.972	≥ 0.979	≥ 0.965	≥ 0.974	≥ 0.981
									
EG									
cFDR	0.249	0.183	0.132	0.112	0.066	0.037	0.094	0.051	0.026
z	4.27	4.52	4.76	4.5	4.78	5.05	4.55	4.85	5.13
n	264,615	123,211	57,584	246,027	117,761	57,227	50,828	23,960	11,822
|r_e1g2_| |r_e1g2·g1_|	≥ 0.836	≥ 0.862	≥ 0.884	≥ 0.847	≥ 0.874	≥ 0.896	≥ 0.848	≥ 0.876	≥ 0.899
EE									
cFDR	0.091	0.054	0.031	0.056	0.026	0.011	0.05	0.021	0.008
z	4.49	4.77	5.03	4.63	4.95	5.26	4.65	4.98	5.32
n	1,028,385	489,485	242,567	1,110,763	535,536	263,301	215,750	106,617	52,114
|r_e1e2_| |r_e1e2·g1_| |r_e1e2·g2_| |r_e1e2·g1g2_|	≥ 0.720	≥ 0.757	≥ 0.788	≥ 0.699	≥ 0.741	≥ 0.778	≥ 0.690	≥ 0.733	≥ 0.773

The sparseness is the percentage of true links among all possible node pairs. Note that the number of declared links is very sensitive to the conditional FDR threshold. For example, for the GG network of human, when the conditional FDR (cFDR) changes from 4.42 to 4.61 (a 4% increase), the number of GG links changes from 13,552 to 6,523 (a 48% decrease). Meanwhile, the sparseness is from 0.02% to 0.01% (a 50% decrease).

### Gene-gene, exon-gene and exon-exon links for human, mouse and rat

To study alternative splicing regulatory networks, we considered exon array data for human, mouse, and rat. For each organism, RNA samples from 11 tissues were profiled using Affymetrix exon arrays. The raw data were downloaded from the Affymetrix website [29] and the gene-level and the exon-level expressions were summarized using Affymetrix Power Tools.

GG association is the traditional GG co-expression association. EG association can be treated as the association between an alternatively spliced exon and its upstream regulators or its downstream target genes, which may not necessarily be direct regulators or direct target genes. Sophisticated models incorporating additional experiments (for example, CLIP experiments) are needed to infer the direct regulators or targets. EE association can be treated as the association between two alternatively spliced exons. The two exons could be regulated by the same direct or indirect splicing regulators. Another scenario could be that a specific transcript isoform of gene 1, which uniquely contains alternative exon 1 compared to other transcript isoforms of gene 1, regulates the exon of gene 2. The latter case is a special exon-transcript association and 'transcript' here represents a particular transcript isoform instead of a family of gene splice variants. The above possible regulation relationships for EG and EE links are diagrammed in Additional data file 2. Additional data file 3 shows the Venn diagram of gene pairs with GG, EG, or EE associations. If GG links mainly reflect the transcriptional regulatory network whereas EG and EE links mainly reflect the alternative splicing regulatory network, it shows that these two networks are largely independent of each other.

### Annotated alternative exons tend to have more exon-gene and exon-exon links

**Figure 2:**
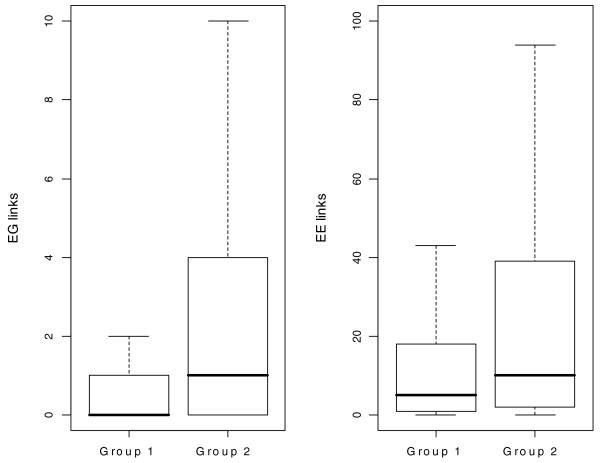
**Boxplot of node degree of constitutive exons and alternative exons**. Two groups of exons were assembled according to transcript annotations from 14 sources. Group 1 represents constitutive exons. Group 2 represents alternative exons. The boxplots of EG links and EE links are plotted (outliers are not drawn). Notice that alternative exons tend to have more EG and EE links than constitutive exons.

If an exon has association links with other exons or genes and such correlations are not due to the GG association, this exon is expected to be an alternatively spliced exon. Otherwise, if the exon is a constitutive exon that has a similar expression level to its gene, the EE or EG correlation is due to the GG correlation. We are interested to know whether EG or EE links can reflect the alternative splicing status of exons. Using the human data as an example, non-redundant transcript annotations were assembled from 14 sources (see details in Materials and methods). These transcripts may be experimentally verified or just computationally predicted. Two groups of exons were then assembled from the large pool of transcript annotations: exons that are present in ≥ 14 transcript isoforms and are not spliced out in any transcript isoform; exons that are present in ≥ 7 transcript isoforms and are spliced out in another ≥ 7 transcript isoforms. The first exon group can be treated as constitutive exons and the second exon group can be treated as alternative exons. Figure 2 shows boxplots of the EG and EE links that the two groups of exons have; exons in group 2 clearly have more EG and EE links than exons in group 1. Specifically, for exons in group 1, 12% have ≥ 5 EG links and 11% have ≥ 50 EE links. For exons in group 2, the percentages increase to 23% for EG links and 21% for EE links. One-sided Wilcoxon tests show that exons in group 2 tend to have more EG and EE links with *p*-values < 2.2 × 10^-16^.

### Conservation of exons with exon-gene or exon-exon links

**Figure 3:**
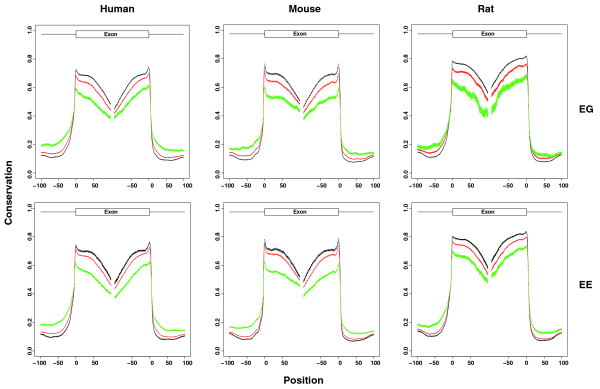
**Conservation of exons with or without EG and EE links**. For every site of an exon, x is defined as the position relative to the nearest splice site. It is positive for distances from the 5' edge and negative for distances from the 3' edge. The upstream intronic region is from -100 to 0 bp and the downstream intronic region is from 0 to 100 bp. Exons were divided into three groups: exons with node degree = 0 (black lines); exons with node degree > 0 and the node degree is in the top 10% of all non-zero node degrees (green lines); exons with node degree > 0 and the node degree is not in the top 10% list (red lines). The y-axis is the average conservation score for the three exon groups. The error bar indicates the standard error of the mean for each position.

It has been reported that the conservation level of alternative exons is lower than that of constitutive exons [30]. On the contrary, the intronic regions flanking alternative exons are more conserved than those flanking constitutive exons [30,31]. To assess whether exons with links to other exons (or genes) tend to be alternatively spliced, we plotted the conservation scores of exons and their flanking regions (Figure 3). Exons were divided into three groups: exons with node degree = 0 (black lines); exons with node degree > 0 and the node degree is in the top 10% of all non-zero node degrees (green lines); exons with node degree > 0 and the node degree is not in the top 10% list (red lines). Node degree is defined as the number of links that a node has to other nodes in the network. Here it represents the number of links that an exon has to other genes (EG) or exons (EE). The average PhastCons conservation score at each exon and flanking region position was calculated and plotted for the three exon groups. Exons with EG or EE links tend to be less conserved than exons without EG or EE links. The flanking intronic regions of exons with EG or EE links tend, however, to be more conserved than those of exons without EG or EE links, which is possibly related to the enriched *cis*-splicing regulatory elements in intronic regions. The more links an exon has, the less it is conserved and the more its flanking intronic regions are conserved. For Affymetrix exon arrays, an exon may represent a cluster of overlapping exons from transcript isoforms with different 5' or 3' splicing sites. The boundary of such an exon cluster may not be the real boundary of the exon in a cell. To eliminate this bias, we removed exons with more than one probe selection region (that is, exons with more than one pair of splicing sites). The results are similar (data not shown).

### Relative position of exons with exon-gene or exon-exon links

**Figure 4:**
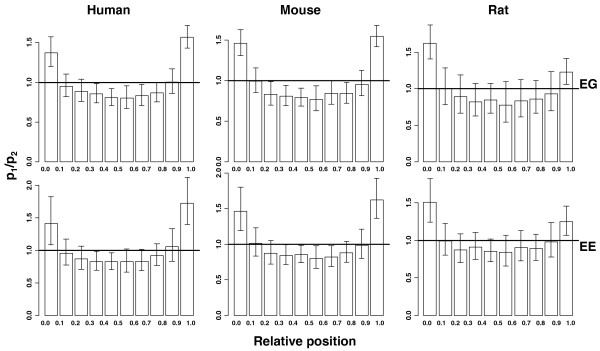
**Enrichment of exons with EG or EE links at the termini of genes**. For each gene, all of the core exons were sorted according to their genomic coordinates (from 5' to 3'). The relative position of the i-th exon is calculated as (i - 1)/(n - 1), where n is the total number of exons. The relative positions were partitioned into ten windows. The proportion of exons with relative positions falling in each window was counted for exons with links and exons without links and denoted as p_1 _or p_2_, respectively. The y-axis represents the p_1_/p_2 _ratio. Error bars represent the 95% confidence intervals of p_1_/p_2_. Notice that p_1_/p_2 _is higher near the terminal regions.

The relative position from 5' to 3' was calculated for each exon, ranging from 0 to 1. The relative positions were partitioned into 10 windows. The proportion of exons with relative positions falling in each window was counted for exons with or without EG (EE) links and denoted as p_1 _or p_2_, respectively. Figure 4 plots the ratio between p_1 _and p_2 _for each relative position window. It clearly shows that exons with EG or EE links tend to be enriched in the initial or terminal regions. Alternative promoters and alternative polyadenylation sites are two of the most prevalent mechanisms for generating transcript isoforms by including alternative first or last exons. Recent studies suggest that 30-50% of human and approximately 50% of mouse genes have multiple alternative promoters [32-36]. In addition, about 54% of human and 32% of mouse genes have alternative polyadenylation sites [37]. Exons with links to other genes or other exons are very likely to be alternatively spliced. Many of them, therefore, are close to the initial or the terminal regions of genes.

### Functional annotation analysis of hubs

We assembled exons with node degrees ranking in the top 1% in the EG network or the EE network. The DAVID functional annotation tool [38] was used with genes to which hub exons belong. The same was done for genes with node degrees ranking in the top 1% in the GG network. Table 2 lists the enriched annotation terms with at least five gene counts, with *p*-values after Bonferroni's correction ≤ 0.001, and that appear at least twice in the nine groups (EG, EE, and GG for human, mouse, and rat). Bonferroni's correction is a very stringent multiple comparison correction. Here it restricts the probability of having one or more falsely declared significant annotation terms to ≤ 0.001. The term 'alternative splicing' is a UniProt knowledgebase keyword meaning 'protein for which at least two isoforms exist due to distinct pre-mRNA splicing events'; it is enriched in genes with hub exons for all of the EG and EE networks. The Uniprot sequence feature 'splice variants' is also enriched in these hub exons. However, 'alternative splicing' and 'splice variants' are not enriched in the gene hubs of the GG networks.

**Table 2:** Functional annotation analysis of exon hubs or gene hubs

		Corrected *p*-value for hubs of EG networks			Corrected *p*-value for hubs of EE networks			Corrected *p*-value for hubs of GG networks		
				
Category	Term	Human	Mouse	Rat	Human	Mouse	Rat	Human	Mouse	Rat
SP	Alternative splicing	2.4 × 10^-13^	6.3 × 10^-25^	2.6 × 10^-10^	1.0 × 10^-21^	5.3 × 10^-20^	3.0 × 10^-19^	N	N	N
UP	Splice variant	1.7 × 10^-06^	5.2 × 10^-14^	5.3 × 10^-05^	8.4 × 10^-12^	4.9 × 10^-08^	1.6 × 10^-11^	N	N	N
MF	Binding	2.3 × 10^-05^	8.0 × 10^-05^	N	1.8 × 10^-07^	3.1 × 10^-06^	3.1 × 10^-04^	N	N	N
SP	Phosphoprotein	1.4 × 10^-17^	8.9 × 10^-09^	N	1.7 × 10^-16^	2.0 × 10^-07^	5.3 × 10^-07^	N	N	N
MF	Protein binding	2.2 × 10^-08^	2.7 × 10^-04^	N	8.0 × 10^-08^	1.1 × 10^-04^	1.7 × 10^-07^	N	N	N
CC	Intracellular	2.6 × 10^-07^	1.0 × 10^-15^	N	1.2 × 10^-20^	2.7 × 10^-06^	N	N	N	N
CC	Intracellular part	8.6 × 10^-09^	6.1 × 10^-13^	N	9.4 × 10^-20^	9.0 × 10^-07^	N	N	N	N
SP	Cytoplasm	N	2.6 × 10^-08^	N	N	6.4 × 10^-08^	N	5.8 × 10^-04^	N	N
CC	Cytoplasm	5.3 × 10^-04^	5.5 × 10^-11^	N	N	2.6 × 10^-07^	N	N	N	N
CC	Intracellular organelle	2.2 × 10^-05^	N	N	9.4 × 10^-18^	N	N	N	6.5 × 10^-04^	N
CC	Organelle	2.4 × 10^-05^	N	N	1.0 × 10^-17^	N	N	N	6.7 × 10^-04^	N
SP	Coiled coil	N	1.6 × 10^-05^	N	N	8.2 × 10^-06^	N	N	N	N
BP	Cellular component organization and biogenesis	N	N	N	2.5 × 10^-04^	N	3.8 × 10^-04^	N	N	N
CC	Intracellular organelle part	N	N	N	4.6 × 10^-0^5	N	N	N	1.9 × 10^-04^	N
BP	Macromolecule metabolic process	5.9 × 10^-05^	N	N	2.5 × 10^-08^	N	N	N	N	N
CC	Nucleus	N	N	N	1.2 × 10^-14^	N	N	N	1.3 × 10^-06^	N
SP	Nucleus	N	N	N	1.9 × 10^-10^	N	N	N	8.1 × 10^-05^	N
CC	Organelle part	N	N	N	5.9 × 10^-05^	N		N	2.0 × 10^-04^	N
CC	Synapse	N	N	N	N	3.0 × 10^-04^	1.1 × 10^-15^	N	N	N
BP	Transport	N	N	9.7 × 10^-04^	N		7.1 × 10^-14^	N	N	N

The DAVID functional annotation tool was applied to genes whose exons are the hubs of EG networks, genes whose exons are the hubs of EE networks, and genes that are the hubs of GG networks. The listed gene annotation terms have at least five gene counts, have *p*-values after Bonferroni's correction ≤ 0.001, and appear at least twice in the nine groups (EG, EE, and GG for human, mouse, and rat). 'N' means the term is not significant for this group. The annotation terms considered here are from the default settings. SP, SP_PIR_KEYWORDS where PIR means protein information resource. UP, UP_SEQ_FEATURE, which means Uniprot sequence feature. BP, GOTERM_BP_ALL where BP means biological process. CC, GOTERM_CC_ALL where CC means cellular component. MF, GOTERM_MF_ALL where MF means molecular function.

### Experimental validation

**Figure 5:**
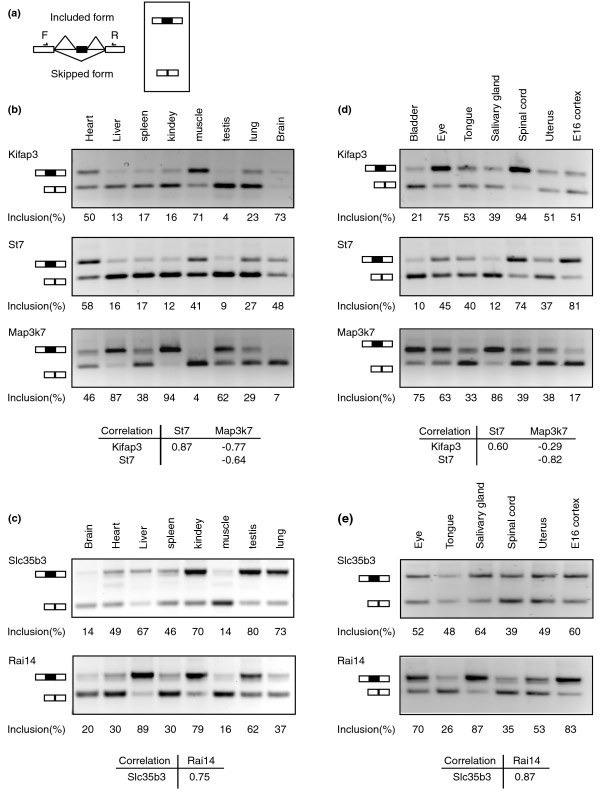
**Examples of EE links illustrated by RT-PCR of tissue RNAs**. **(a) **Scheme of RT-PCR design to examine splicing levels of alternative exons. Primers (arrows) are in the flanking constitutive exons. Inclusion levels of alternative exons (black box) are calculated as Included form/(Included form + Skipped form). **(b)**. Alternative splicing of *Kifap3 *exon20, *St7 *exon 7, and *Map3k7 *exon 12 in multiple mouse tissues. *Kifap3 *exon 20 is positively correlated with *St7 *exon 7 and negatively correlated with *Map3k7 *exon 12. *St7 *exon 7 is negatively correlated with *Map3k7 *exon 12. Pair-wise Pearson correlations based on the RT-PCR experiments are shown. **(c) **Slc35b3 exon 4 is positively correlated with Rai14 exon 11. **(d) **Pair-wise correlations between *Kifap3 *exon20, *St7 *exon 7, and *Map3k7 *exon 12 in a second set of tissues not surveyed by the Affymetrix exon array. **(e) **Pair-wise correlation between *Slc35b3 *exon 4 and *Rai14 *exon 11. Percentages of inclusion levels were averaged from three independent experiments.

We experimentally examined the pCastNet results by RT-PCR across various tissues. In particular, the EE link is a relatively new correlation subject (in biology) and a very interesting phenomenon. We randomly chose EE links at the lower bound of the correlation cut-off (about 0.75-0.80) but favored cassette exons because of the ease of RT-PCR design. Due to the nature of our data, we also favored genes that are expressed in multiple tissues in order for PCR to amplify with the same number of cycles across the tissues. pCastNet found significant EE links among Kinesin-associated protein 3 (*Kifap3*) exon 20 (exon id 24930 in Affymetrix exon array), Suppression of tumorigenicity 7 (*St7*) exon 7 (exon id 685163), and Mitogen-activated protein kinase kinase kinase 7 (*Map3k7*) exon 12 (exon id 572746). For convenience, we refer to these exons as Kifap3_20, St7_7, and Map3k7_12. Specifically, pCastNet predicted that Map3k7_12 has negative associations with both Kifap3_20 and St7_7 (correlations and partial correlations are about -0.75 for both), whereas Kifap3_20 has a positive association with St7_7 (correlation and partial correlations are about 0.80). NCBI EST database shows these exons are all alternative exons. Primers were designed in the flanking constitutive exons to amplify transcripts either containing or skipping these alternative exons. RT-PCR results and Pearson correlation analysis of exon inclusion levels (Figure 5b) show that Map3k7_12 is negatively correlated with both Kifap3_20 and St7_7 while Kifap3_20 is positively correlated with St7_7 in these tissues. Besides the tissues surveyed in the exon array study, we also performed RT-PCR experiments in seven other tissues (Figure 5d). Based on the RT-PCR experiments, the correlation between Kifap3_20 and St7_7 is 0.60 and the correlation between Map3k7_12 and St7_7 is -0.82 whereas the correlation between Map3k7_12 and Kifap3_20 dropped to -0.29. Another example is a positive correlation between Solute carrier family 35, member B3 (*Slc35b3*) exon4 (exon id 226950, or Slc35b3_4) and Retinoic acid induced 14 (*Rail4*) exon 11 (exon id 300782, or Rai14_11). pCastNet predicts a positive association between these two exons (correlation and partial correlations are about 0.80). RT-PCR and Pearson correlation analysis (Figure 5c) show a positive correlation of 0.75 among the tested tissues used by the Affymetrix exon array. In the second set of tissues, RT-PCR experiments show that their correlation is about 0.87 (Figure 5e). Note that Slc35b3 is not detectable in bladder, and thus has not been included in the correlation analysis.

### Functional similarity of gene pairs with links

All of the above results indicate that pCastNet can effectively identify EG and EE links. We then further explored the possible functional relationship between two genes with an EG link or an EE link. Using the human data and the Molecular Signatures Database [39], genes were grouped into gene sets according to: their chromosome positions; curated information from pathway databases; shared conserved *cis*-regulatory motifs; and shared Gene Ontology (GO) terms. We tested whether genes with EG or EE links tend to be in the same gene sets using hypergeometric tests. The results are summarized in Table 3. Genes in the same chromosomal cytogenetic band ('c1') are more likely to have GG and EG links than EE links. Gene pairs with GG, EG, or EE links tend to be in the same pathways (these pathways are collected by the BioCarta, GenMAPP, and KEGG databases). More interestingly, gene pairs with EG or EE links tend to be in the same motif gene sets ('c3'). Specifically, genes in those sets share a motif in the promoter regions ('c3_promoter_known' and 'c3_promoter_unknown') or a microRNA (miRNA) binding site in the 3' untranslated regions ('c3_miRNA'). On the contrary, the *p*-values of GG links in the promoter motif sets are less significant than those of EG and EE links. And gene pairs with GG links are not enriched in the 'miRNA binding' gene sets. In addition, exons with EE links and sharing miRNA binding motifs tend to be enriched at the 3' terminals of the genes (Additional data file 4). Finally, genes with GG, EG or EE links all tend to share GO terms.

**Table 3:** Gene pairs sharing gene sets and having GG, EG, or EE links

		Gene pairs having GG links (13,552)		Gene pairs having EG links (223,116)		Gene pairs having EE links (815,024)	
				
Gene set category	No. of gene pairs sharing a gene set among a total of 53,721,795 gene pairs	No. of gene pairs also sharing gene set	*p*-value	No. of gene pairs also sharing gene set	*p*-value	No. of gene pairs also sharing gene set	*p*-value
c1	321,284	150	2.1 × 10^-12^	1,584	1.1 × 10^-11^	4,855	0.61
c2_BioCarta	30,023	40	2.0 × 10^-17^	238	5.4 × 10^-20^	601	2.3 × 10^-11^
c2_GenMAPP	49,570	101	2.4 × 10^-56^	421	4.4 × 10^-40^	1,085	6.1 × 10^-31^
c2_KEGG	182,069	348	1.1 × 10^-179^	1,137	1.1 × 10^-38^	3,048	3.1 × 10^-08^
c3_miRNA	2,373,102	556	0.96	12,477	8.7 × 10^-150^	49,006	< 4.9 × 10^-324^
c3_promoter_known	14,479,685	4,207	1.7 × 10^-26^	67,435	7.5 × 10^-261^	248,414	< 4.9 × 10^-324^
c3_promoter_unknown	3,186,951	915	3.5 × 10^-05^	14,507	1.1 × 10^-29^	55,308	1.1 × 10^-227^
c4_bp	7,107,791	2,947	5.5 × 10^-163^	35,289	6.9 × 10^-272^	129,574	< 4.9 × 10^-324^
c4_cc	6,549,156	4,418	< 4.9 × 10^-324^	35,702	< 4.9 × 10^-324^	127,572	< 4.9 × 10^-324^
c4_mf	815,318	581	3.7 × 10^-104^	4,294	4.3 × 10^-52^	15,897	1.0 × 10^-209^

Among the 10,366 human genes that were considered, there are 53,721,795 possible gene pairs. About 13,552 gene pairs were declared to have GG links; 223,116 gene pairs were declared to have at least one EG link; and 815,024 gene pairs were declared to have at least one EE link. The number of gene pairs that have GG, EG, or EE links and are in the same gene set is listed. The *p*-value of observing such a high or higher number of gene pairs that have GG, EG, or EE links and are in the same gene set was based on a hypergeometric test. Different gene set categories were considered. 'c1', genes sharing chromosomal cytogenetic bands; 'c2_BioCarta', 'c2_GenMAPP', and 'c2_KEGG', genes in the same pathways and the pathways were collected from the BioCarta, GenMAPP, or KEGG databases; 'c3_miRNA', genes sharing a microRNA binding site; 'c3_promoter_known' and 'c3_promoter_unknown', genes sharing a motif in the promoter regions and the motif matches a known transcription factor binding site or the motif does not match any known transcription factor binding site; 'c4_bp', genes sharing biological process ontology terms; 'c4_cc', genes sharing cellular component ontology terms; 'c4_mf', genes sharing molecular function ontology terms.

**Figure 6:**
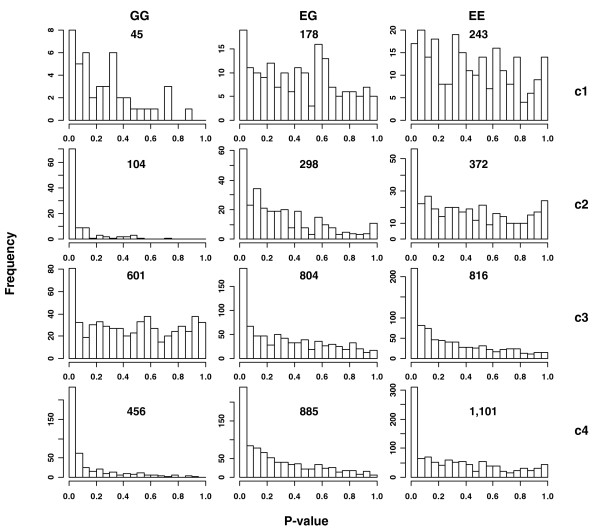
**Enrichment of GG, EG, or EE links in functional gene sets**. For each gene set with at least one GG, EG, or EE link, to test the significance of the enrichment of links, we simulated gene sets by randomly selecting the same number of genes as the tested gene set. The empirical *p*-value of the number of observed links was calculated as Pr (the number of links in the simulated gene set ≥ the number of observed links) from 1,000 simulations. Histograms of the *p*-values are plotted for those gene sets. The total number of test gene sets is listed on the histograms. C1: gene sets sharing a chromosomal cytogenetic band. C2: gene sets curated from pathway databases. C3: gene sets sharing a conserved *cis*-regulatory motif. C4: gene sets sharing a GO term.

We also examined *p*-values for the enrichment of links in each individual gene set. We counted the number of GG, EG, or EE links between members of a gene set for each gene set. To test the significance of the enrichment of links, we simulated gene sets by randomly selecting the same number of genes. The simulated gene sets have no functional similarity. We then calculated the empirical *p*-values of the number of observed GG, EG or EE links as Pr (the number of links in the simulated gene set ≥ the number of observed links) from 1,000 simulations. Figure 6 plots the histogram of the *p*-values of gene sets with at least one observed GG, EG, or EE link. For all gene set categories except category 1, there are more gene sets enriched with GG, EG or EE links compared with the random selections, where a uniform distribution of *p*-values is expected.

### Examples

The motif (U)GCAUG has been reported as a binding motif for mammalian splicing factors FOX-1 (A2BP1) and FOX-2 (RBM9) [40-43]. We studied the enrichment of motif GCAUG in exons with EG links to FOX-1 and FOX-2. For each exon, we counted the occurrence of the pentamer GCAUG in the exonic region and the flanking 200 bp intronic regions. Table 4 shows the enrichment of this motif for exons correlated with FOX-1 or FOX-2. The empirical *p*-values of the enrichment were based on 1,000 simulated exon groups. Among the 64 exons correlated with FOX-1 in human, GCAUG occurs 96 times. And none of the 1,000 simulated exon groups has more than 96 occurrences of GCAUG. The expression level of FOX-1 and the inclusion rates of its associated exons are plotted in Additional data file 5; this clearly shows the co-expression patterns between FOX-1 and exons with EG links to FOX-1. Although the *p*-values for FOX-2 in human (0.031) and Fox-1 and Fox-2 in mouse (0.172, 0.060) are less significant, the occurrences of GCAUG are about twice as many as the average occurrence among the 1,000 simulated groups. Note that after the filtering procedures for the raw data, FOX-1 and FOX-2 are not in the final gene list for rat.

**Table 4:** Motif enrichment for genes with EG links to FOX-1 and FOX-2

	Human		Mouse	
		
	FOX-1(A2BP1)	FOX-2(RBM9)	Fox-1(A2bp1)	Fox-2(Rbm9)
Number of exons with EG links to FOX-1 or FOX-2	64	21	19	5
Number of GCAUG occurrences among exons with EG links to FOX- 1 or FOX-2	96	28	21	9
Average number of GCAUG occurrences among 1,000 simulated exon groups	49.4	16.6	15.8	4.2
*P*-value of the motif enrichment	0.000	0.031	0.172	0.060

Exons with EG links to FOX-1 or FOX-2 were assembled. The occurrences of GCAUG were calculated for those exon groups. To test the significance of the motif enrichment, we simulated exon groups by randomly selecting exons. The *p*-value of the motif enrichment was calculated as Pr (the motif frequency in the simulated exon group ≥ the observed motif frequency) from 1,000 simulations.

**Figure 7:**
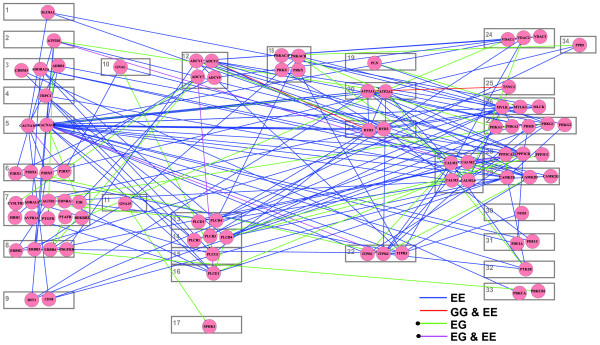
**GG, EG, and EE links in the calcium signaling pathway**. The gene layout is the same as the layout in the KEGG pathway database. A red line represents a GG link. The two gene pairs with GG links happen to also have EE links ('GG & EE'). A blue line represents an EE link and a green line represents an EG link. A dot at one end of a line is used to represent the exon in an EG link. The KEGG calcium signaling pathway is enriched with EG and EE links with *p*-values 0.01 and 0.001. However, GG links are not enriched in the pathway, with a *p*-value of 0.354. Each box represent the corresponding component in the KEGG database: 1, NCX; 2, PMCA; 3, GPCR; 4, SOC; 5, CaV1; 6, ROC; 7, GPCR; 8, PTK; 9, CD38; 10, Gs; 11, Gq; 12, ADCY; 13, PLCδ; 14, PLCβ; 15, PLCγ; 16, PLCε; 17, SPHK; 18, PKA; 19, PLN; 20, SERCA; 21, RYR; 22, IP3R; 23; CALM; 24, VDAC; 15, TnC; 26, MLCK; 27, PHK; 28, CaN; 19, CAMK; 30, NOS; 31, PDE1; 32, FAK2; 33, PKC; 34, PPID. A circle in a box represents a gene in the corresponding component.

The calcium signaling pathway has been shown to be intensively related to alternative splicing [44]. In our gene set analysis, the KEGG calcium signaling pathway is enriched with EG and EE links with empirical *p*-values of 0.01 and 0.001, respectively. However, GG links are not enriched in the pathway, with a *p*-value of 0.354. Figure 7 plots the EG, EE, and GG links in the pathway. The gene layout is the same as the layout in the KEGG database. Most components of the calcium signaling pathway from the KEGG database have at least one link, as shown in Figure 7. Red lines represent GG links (the two gene pairs happen to have EE links also). Blue links represent EE links and green lines represent EG links. The above results indicate the important role of alternative splicing in signaling pathways and/or the important roles of calcium signaling pathways in alternative splicing regulation.

## Discussion

In this paper, we propose the use of pCastNet to identify EE co-splicing links and EG co-expression links. pCastNet avoids taking the ratio between exon-level intensity and gene-level intensity and it achieves a higher statistical power compared to an NI-based approach (Figure 1). Such EG and EE links can provide information about alternative splicing. For example, alternative exons have significantly more EG or EE links than constitutive exons (Figure 2). Secondly, exons with EG or EE links tend to be less conserved in exonic regions than exons without EG or EE links. On the contrary, the flanking intronic regions of exons with EG or EE links tend to be more conserved than those of exons without EG or EE links (Figure 3). Such observations are consistent with the conservation patterns of alternative exons and constitutive exons [30]. In addition, exons with EG or EE links tend to be enriched in the 5' or 3' termini of genes where alternative splicing events are enriched (Figure 4). The functional annotation analysis also indicates that genes containing exon hubs of EG or EE networks tend to have multiple splicing isoforms (Table 2). All the results indicate that the EG or EE links can reflect the alternative splicing status of exons. Furthermore, they can provide information about the alternative splicing regulatory network.

The alternative splicing regulatory network reconstructed by pCastNet is composed of nodes (exon or gene) and their pair-wise association links. It provides a different way to study alternative splicing from previous differential analysis. Typical differential analysis compares two tissues or conditions; for example, by studying differential alternative splicing between tissues, one can identify a cluster of tissue-specific alternative splicing events. By studying differential alternative splicing after the knockdown of splicing factors, one can identify a cluster of target candidates of splicing regulators. pCastNet considers multiple conditions at one time; by studying co-expression patterns of nodes across multiple conditions, we can identify pair-wise links between nodes. From these links regulatory or functional relationships can be inferred and they provide a comprehensive view of alternative splicing regulation. However, links identified by the current study are association links and not necessarily causal links. The possible regulatory relationships they reflect can be direct or indirect. CLIP based and knockdown experiments are more powerful tools to identify direct causal links. Although pCastNet does not provide as strong evidence as CLIP studies for identifying downstream targets of splicing factors, it infers other spaces of regulation, for example, upstream regulatory genes besides the splicing factors of interest. If one is interested in a specific signaling pathway where multiple components can affect each other simultaneously, pCastNet can identify invaluable links to dissect the regulatory relationship. As we show in the calcium signaling pathway example, EE and EG links but not GG links are significantly enriched and the results provide clues to investigate the functional and regulatory relationships between nodes. In summary, pCastNet and differential study are complementary to each other and should be considered in combination to better understand the network of interest.

We validated pCastNet predictions using RT-PCR experiments. No studies have reported the co-splicing of exons Kifap3_20, St7_7, and Map3k7_12 nor reported on the functional relationships between Kifap3, St7, and Map3k7. Kifap3 is an auxiliary factor of the Kinesin family member 3 (*Kif3*) heterotrimer complex that links KIF3 with its cargos [45]. Alternative splicing of Kifap3_20 generates two Kifap3 isoforms that differ in the carboxyl terminus region. *St7 *has been reported to be a tumor suppressor gene involved in multiple types of cancer [46]. In addition, Vincent *et al. *[47] reported that *St7 *spans a translocation point in a patient with autism. They also observed the alternative splicing of exon 7. Map3k7 is a mitogen-activated protein kinase kinase kinase that transduces intracellular signals from the interleukin-1 receptor [48] and tumor necrosis factor receptor [49]. Kondo and colleagues [50] reported strong biases of isoform (either including or excluding Map3k7_12) ratios in some lung cancer specimens. Why and how the splicing of Kifap3_20, St7_7, and Map3k7_12 are coordinated in some tissues is an interesting question.

The selection of samples or conditions will impact the identification and biological interpretation of links. Although we had preferred to study a group of phenotypically relevant conditions (for example, different parts of the brain) to better infer the biological meanings of links, there were limited data available at the time of our study. As our data are from diverse tissues, the network more likely identifies links shared by the majority of the selected tissues. For example, pCastNet predicts a significant correlation between exon Slc35b3_4 and exon Rai14_11. RT-PCR experiments have validated the correlation in tissues of brain, heart, liver, spleen, kidney, muscle, lung and testis (Figure 5c). Besides these eight tissues surveyed by the Affymetrix exon array, we also considered a different set of tissues not surveyed by the exon array study to see if this correlation is a general pattern extendable to other tissues. RT-PCR clearly shows the positive correlation among eye, tongue, salivary gland, spinal cord, ovary and E16 cortex (Figure 5e). This is consistent with the idea that due to the sources of our data, links identified by pCastNet in the current study tend to be a general phenomenon shared by multiple tissues. Another example is the pair-wise correlations between the splicing of Kifap3_20, St7_7, and Map3k7_12 in brain, heart, liver, spleen, kidney, muscle, lung and testis (Figure 5b). For those seven additional tissues, Kifap3_20 and St7_7 are still positively associated, with a correlation of 0.60. Map3k7_12 and St7_7 are still negatively associated with a correlation of -0.82. However, the negative correlation between Map3k7_12 and Kifap3_20 decreases to -0.29 (Figure 5d). Therefore, although these exons are coordinately regulated in general, such a relationship remains context-dependent. If their correlation is directly caused by one or a few alternative splicing regulators, we could surmise that the tissue-specific expression of these splicing regulators and their differential *trans*-activity strength on the three exons confers the context-dependent correlation. Another explanation would be that the three exons have separate unique regulators besides the common regulators. The unique regulators counteract the effects of common regulators and are expressed in a tissue-specific manner. In the future, the power of pCastNet will be extended by combining it with transcriptome differential analysis and RNA binding protein motif analysis in order to elucidate the coordinate and combinatorial alternative splicing regulatory network.

We discovered the functional similarity of gene pairs with EG or EE links. Strikingly, gene pairs with EG or EE links tend to share a conserved sequence element in their promoter regions. However, the *p*-values for gene pairs with GG links are less significant. It has been reported and remains a puzzle that, in mammals, the direct correlation between regulatory *cis*-element similarity and expression similarity is not significant [51]. A second striking phenomenon is that gene pairs with EG or EE links tend to share miRNA targets (Table 3). However, the *p*-value for GG links is not significant, which is consistent with the general concept that miRNAs mainly affect protein translation but not transcript amounts in mammals. These results indicate the coupling of co-alternative splicing, co-transcription factor binding, and co-miRNA binding for a pair of genes. For example, genes sharing transcription factor binding sites may have co-regulated alternative promoters, which leads to the coupling of co-transcription-factor binding and co-alternative splicing. Besides alternative promoters, downstream alternative exons may also be involved in the coupling because alternative promoters have been reported to be correlated with downstream alternative splicing [52-56]. Thus, the transcription factor binding may be associated with the choice of promoters, while the choice of promoter again is associated with the inclusion or exclusion of a downstream alternative exon. Another explanation is that the conserved sequence elements could be RNA *cis-*elements for alternative splicing regulation instead of DNA *cis-*elements for transcription regulation because the considered promoter region is large (covering -2 kb to 2 kb around transcription start sites). Future work will need to explore the detailed mechanisms. The enrichment of EG and EE links for genes in the same pathways or having the same GO terms also suggest that we can predict gene functions by considering neighboring genes in splicing regulatory networks.

Several groups published a few sets of transcriptome data from high-throughput sequencing techniques while this manuscript was under preparation. Such techniques improve the accuracy of expression level measurement and increase the efficiency of identifying novel alternative exons as exon junctions are sequenced. Our proposed methods can be directly applied on transcriptome RNA-Seq data to identify EE and EG links more accurately.

## Conclusion

We propose a partial correlation analysis approach, pCastNet, to reconstruct EE and EG networks. EE and EG networks are part of alternative splicing regulatory networks. We confirmed that pCastNet can effectively identify EG and EE links through studying known alternative exons, conservation patterns, relative positions, and functional annotations, and by RT-PCR experiments. We also found that genes with EG or EE links with each other tend to have similar functions or are in the same pathways, and genes with EG or EE links tend to share *cis*-regulatory motifs in promoter regions and 3' untranslated regions. Through these networks, we can gain a better understanding of the role of alternative splicing in the gene regulatory network.

## Materials and methods

### Exon array pre-processing

Exon array (Human Exon 1.0 ST, Mouse Exon 1.0 ST, Rat Exon 1.0 ST) data for 11 tissues were downloaded from the Affymetrix website [29]. The profiled tissues for human include breast, cerebellum, heart, kidney, liver, muscle, pancreas, prostate, spleen, testis, and thyroid. The tissues for mouse and rat include brain, embryo, heart, kidney, liver, lung, muscle, ovary, spleen, testicle, and thymus. There are three replicates for each tissue. The probe intensities were quantile normalized and were adjusted based on the median intensity of probes with similar GC content. The PLIER method was used to summarize the probe-set-level intensity. The iter-PLIER method was used to summarize the gene-level intensity by iteratively calling PLIER with the core probe sets (that is, RefSeq supported) that correlate with signal estimates. In the design of Affymetrix exon arrays, gene annotations from databases were projected onto the genome to infer transcript clusters and exon clusters. A transcript cluster roughly corresponds to a gene. In many cases, an exon cluster represents a true biological exon and it acts as one probe selection region. In other cases, an exon cluster represents the union of multiple overlapping exons possibly due to alternative splice sites. Such exon clusters were further fragmented into multiple probe selection regions according to the hard edges (for example, splice sites). In exon array, hard edge was defined as the end of the sequence that defines the boundary of a probe selection region and cannot be extended beyond the border by other annotation evidence. The probe set annotation, the transcript cluster annotation, and the exon annotation were downloaded form the Affymetrix website (version hg18 for human, mm9 for mouse, and rn4 for rat) [29]. To avoid knowledge bias, only core exons based on RefSeq transcripts or full-length mRNAs were considered. Note that 'core probe sets' in Affymetrix exon arrays only means that they are supported by RefSeq transcripts or full-length mRNAs; they do not contain any information about whether they are alternative exons or constitutive exons.

Presence or absence of probe sets was determined by a 'detection above background' *p*-value threshold of 0.05. Genes with more than 50% 'present' core probe sets were called 'present'. The following filtering procedures were performed for probe sets: remove probe sets that are not core probe sets; remove probe sets whose genes are present in < 11 arrays out of 33 arrays (11 tissues × 3 replicates) or whose genes are mapped to more than one Entrez gene ID; remove probe sets that are present in < 11 arrays out of 33 arrays; remove probe sets with (Maximum intensity)/(Minimum intensity) < 5. After the above procedures, a total of 97,293 probe sets corresponding to 76,038 exon clusters and 10,366 transcript clusters remain for human; a total of 102,729 probe sets corresponding to 82,145 exon clusters and 10,765 transcript clusters remain for mouse; and a total of 45,691 probe sets corresponding to 40,082 exon clusters and 5,077 transcript clusters remain for rat. The average intensity across the three replicates was log_2 _transformed and used as the intensity level for each tissue.

### Correlation and partial correlation calculation

The Pearson correlation coefficient is denoted as r_ab _between variable a and variable b. The first-order partial correlation coefficient between a and b conditioning on c is [57]:

$${\text{r}}_{\text{ab}•\text{c}}=\frac{{\text{r}}_{\text{ab}}-{\text{r}}_{\text{ac}}{\text{r}}_{\text{bc}}}{\sqrt{\left(1-{\text{r}}_{\text{ac}}^{2})(1-{\text{r}}_{\text{bc}}^{2}\right)}}.$$

The second-order partial correlation coefficient between a and b conditioning on c and d is:

$${\text{r}}_{\text{ab}•\text{cd}}=\frac{{\text{r}}_{\text{ab}•\text{c}}-{\text{r}}_{\text{ad}•\text{c}}{\text{r}}_{\text{bd}•\text{c}}}{\sqrt{\left(1-{\text{r}}_{\text{ad}•\text{c}}^{2})(1-{\text{r}}_{\text{bd}•\text{c}}^{2}\right)}}.$$

Note that r_ab·cd _= r_ab·dc _theoretically (see proof in Additional data file 6). In pCastNet, for GG associations, the Pearson correlation coefficient between gene 1 (g_1_) and gene 2 (g_2_) was calculated as ${\text{r}}_{{\text{g}}_{1}{\text{g}}_{2}}$. For EG associations, the partial correlation coefficient between an exon (e_1_) of gene 1 and gene 2 (g_2_) conditioning on gene1 (g_1_) was calculated as ${\text{r}}_{{\text{e}}_{1}{\text{g}}_{2}•{\text{g}}_{1}}$. The Pearson correlation was calculated as ${\text{r}}_{{\text{e}}_{1}{\text{g}}_{2}}$. For EE associations, the Pearson correlation ${\text{r}}_{{\text{e}}_{1}{\text{e}}_{2}}$ and the partial correlations ${\text{r}}_{{\text{e}}_{1}{\text{e}}_{2}•{\text{g}}_{1}}$, ${\text{r}}_{{\text{e}}_{1}{\text{e}}_{2}•{\text{g}}_{2}}$, and ${\text{r}}_{{\text{e}}_{1}{\text{e}}_{2}•{\text{g}}_{1}{\text{g}}_{2}}$ were calculated according to the above equations.

### Simulation studies

In simulation studies, five genes were considered. Each of them has five constitutive exons and one alternative exon. The intensity data of the five constitutive exons of gene g (g = 1,..., 5) in tissue t (t = 1,..., 10) were simulated according to the normal distribution *N*(μ_gt_, σ^2^), where μ_gt _is a value sampled from the range 4 to 9 and it is different for different genes g and different tissues t. All of the five alternative exons have the same inclusion rate relative to their genes: (τ_1_, τ_2_,..., τ_10_) = (0.1, 0.2,..., 1.0) for the 10 tissues. Thus, the intensity data of the alternative exon of gene g was simulated according to the normal distribution *N*(μ_gt_τ_t_, σ^2^). The gene-level intensity was estimated as the average value of the constitutive exons. pCastNet and the NI-based approach were conducted to calculate the correlations and partial correlations between exons belonging to different genes. Three scenarios were considered (σ = 1, 2, or 4). For each scenario, 1,000 simulations were performed and the average true positive rate and the average false positive rate were calculated according to different correlation thresholds.

### Conditional false discovery rate control

For GG associations, the test statistic:

$$\text{t}={\text{r}}_{{\text{g}}_{1}{\text{g}}_{2}}\sqrt{\left(\text{n}-2)/(1-{\text{r}}_{{\text{g}}_{1}{\text{g}}_{2}}^{2}\right)}$$

is converted to z-value:

z = Φ^-1^(G_0_(t))

G_0_(t) is the null cumulative distribution function for the t-values, Φ^-1 ^is the inverse function of the cumulative distribution function of standard normal, and n is the number of tissues being surveyed. Under the null hypothesis that there is no correlation between gene g_1 _and gene g_2_, t follows a Student t distribution with degrees of freedom n - 2 and z follows the standard normal distribution.

For EG association, the t values are:

$${\text{t}}_{1}={\text{r}}_{{\text{e}}_{1}{\text{g}}_{2}}\sqrt{\left(\text{n}-2)/(1-{\text{r}}_{{\text{e}}_{1}{\text{g}}_{2}}^{2}\right)}$$

and

$${\text{t}}_{2}={\text{r}}_{{\text{e}}_{1}{\text{g}}_{2}•{\text{g}}_{1}}\sqrt{\left(\text{n}-3)/(1-{\text{r}}_{{\text{e}}_{1}{\text{g}}_{2}•{\text{g}}_{1}}^{2}\right)}$$

They are converted to z-values:

z_1 _= Φ^-1^(G_01_(t_1_))

and

z_2 _= Φ^-1^(G_02_(t_2_))

Under the null hypothesis, t_1 _follows a Student t distribution with degrees of freedom n - 2 and t_2 _follows a Student t distribution with degrees of freedom n - 3. The test statistic is:

z_0 _= min(|z_1_|, |z_2_|)

p = pr(Z_0 _≥ z_0_) = pr(|Z_1_| ≥ z_0_, |Z_2_| ≥ z_0_)

Under the null, Z_1 _and Z_2 _follow a bivariate normal distribution with the correlation approximated as the sample correlation between Z_1 _and Z_2 _across different node pairs. The final z-value is:

z = sign (t_1_)Φ^-1^(1 - p/2)

For EE associations, the t values are:

$${\text{t}}_{1}={\text{r}}_{{\text{e}}_{1}{\text{e}}_{2}}\sqrt{\left(\text{n}-2)/(1-{\text{r}}_{{\text{e}}_{1}{\text{e}}_{2}}^{2}\right)}$$

$${\text{t}}_{2}={\text{r}}_{{\text{e}}_{1}{\text{e}}_{2}•{\text{g}}_{1}}\sqrt{\left(\text{n}-3)/(1-{\text{r}}_{{\text{e}}_{1}{\text{e}}_{2}•{\text{g}}_{1}}^{2}\right)}$$

$${\text{t}}_{3}={\text{r}}_{{\text{e}}_{1}{\text{e}}_{2}•{\text{g}}_{2}}\sqrt{\left(\text{n}-3)/(1-{\text{r}}_{{\text{e}}_{1}{\text{e}}_{2}•{\text{g}}_{2}}^{2}\right)}$$

$${\text{t}}_{4}={\text{r}}_{{\text{e}}_{1}{\text{e}}_{2}•{\text{g}}_{2}{\text{g}}_{1}}\sqrt{\left(\text{n}-4)/(1-{\text{r}}_{{\text{e}}_{1}{\text{e}}_{2}•{\text{g}}_{2}{\text{g}}_{1}}^{2}\right)}$$

They are converted to z-values:

z_1 _= Φ^-1^(G_01_(t_1_))

z_2 _= Φ^-1^(G_02_(t_2_))

z_3 _= Φ^-1^(G_03_(t_3_))

z_4 _= Φ^-1^(G_04_(t_4_))

Under the null hypothesis, t_1 _follows a Student t distribution with degrees of freedom n - 2; t_2 _and t_3 _follow a Student t distribution with degrees of freedom n - 3; t_4 _follows a Student t distribution with degrees of freedom n - 4. The test statistic is:

z_0 _= min(|z_1_|, |z_2_|, |z_3_|, |z_4_|)

p = pr(Z_0 _≥ z_0_) = pr(|Z_1_| ≥ z_0_, |Z_2_| ≥ z_0_, |Z_3_| ≥ z_0_, |Z_4_| ≥ z_0_,)

Under the null, Z_1_, Z_2_, Z_3_, Z_4 _follow a multivariate normal distribution with the correlations approximated as the sample correlations between Z_1_, Z_2_, Z_3_, Z_4 _across different node pairs. The final z-value is:

z = sign (t_1_)Φ^-1^(1 - p/2)

For a threshold x, the conditional FDR is estimated as [25]:

$$\text{FDR}(\text{x}|\text{A})=\frac{2\text{N}\Phi (-\text{x})}{\#\{|{\text{z}}_{\text{i}}|\geq \text{x}\}}\left[1+\text{A}\frac{\text{x}\phi (\text{x})}{\sqrt{2}\Phi (-\text{x})}\right]$$

where Φ is the standard normal cumulative distribution function, N is the total number of node pairs, φ is the standard normal density function, and $\text{A}=\frac{2\Phi (1)-1-\#\{{\text{z}}_{\text{i}}\in [-1,1]\}/\text{N}}{\sqrt{2}\phi (1)}$. For a FDR(x|A), the percentage of true links among all possible node pairs is estimated as **#{|z_i_| ≥ x}(1 - FDR(x|A))/N**.

### RNA preparation and RT-PCR

Various tissues from adult C57BL mice and embryonic cortex from E16 embryos were dissected and quickly submerged in Trizol (Invitrogen, Carlsbad, CA, USA) followed by immediate tissue homogenization. Total RNA samples were prepared according to manufacturer's protocol. RT-PCR was done as previously described [30]. Primer design was done with the Primer3 online software [58]. Primer sequences were. *Kifap3*, CCTCCAGAATGGAGATGTGG (forward), ACATGGGAGGGGTGATTTTA (reverse); *St7*, GCAGATGCAATAATGCAAAAAG (forward), GTAACAACCATCTCCAGCCTTC (reverse); *Map3k7*, TCTGAAATAGAAGCCAGGATCG (forward), CTTCTCTGAGGTTGGTCCTGAG (reverse); *Slc35b3*, AGCCTTACGGCTGGTACCTT (forward), AGTTTGGTGCAATTGTGCTG (reverse); *Rai14*, TCTCATGCTGGCTTGTGAAA (forward), GTTATTGATCGTGGGGAGGA (reverse). Identities of each RT-PCR product were confirmed by direct sequencing. PCR bands were quantified using ImageQuant TL software (GE Healthcare Bio-Sciences, Piscataway, NJ, USA). The correlation value of the each exon pair was calculated as the Pearson correlation coefficient between the inclusion levels of the two tested exons. Inclusion levels based on the PCR results were calculated as Inclusion form/(Inclusion form + Exclusion form).

### Other datasets and analysis

Non-redundant human transcript annotations were assembled based on AceView gene [59], AUGUSTUS gene [60], CCDS gene [61,62], Ensembl gene [61], Geneid gene, Genescan gene [63], MGC gene, N-SCAN gene [64], ORFeome gene, RefSeq gene [62], SGP gene, SIB gene [65], UCSC genes [66], and ASTD gene [67]. The first 13 data sources were downloaded from the UCSC Genome Browser website (hg18) [68] and the last was downloaded from the ASTD database (release 1.0) [69]. A gene may have multiple transcript isoforms. For each exon cluster defined by the Affymetrix exon array, if one exon of a transcript isoform locates in the exon cluster region, the exon cluster is called 'present' in this transcript isoform. If the exon cluster locates in an intron region of a transcript isoform, the exon cluster is called 'spliced out' in this transcript isoform. Exon clusters (or exons for simplicity) were divided into two groups: exons that are present in ≥ 14 transcript isoforms and are not spliced out in any transcript isoform; exons that are present in ≥ 7 transcript isoforms and are spliced out in another ≥ 7 transcript isoforms.

The PhastCons conservation score [70] was downloaded from the UCSC Genome Browser (hg18) [68]. The score of each site is the posterior probability that the site is in the conserved state of the phylogenetic hidden Markov model for 17 vertebrates.

For each gene with multiple exons, all of the core exons were sorted according to their genomic coordinates (from 5' to 3'). The relative position of the i-th exon is calculated as (i - 1)/(n - 1), where n is the total number of exons. The relative position ranges from 0 to 1.

Gene sets downloaded from the Molecular Signatures Database [39] belong to four categories: 'c1', positional gene sets for each chromosomal cytogenetic band; 'c2', curated gene sets from pathway databases; 'c3', motif gene sets sharing conserved *cis*-regulatory motifs [71]; 'c4', GO gene sets sharing the same GO term. We removed gene sets without any gene in our final gene list for exon arrays. Category 2 was further divided into 'c2_BioCarta', 'c2_GenMAPP', and 'c2_KEGG'; genes in the same pathways and the pathways were collected from the BioCarta database, the GenMAPP database, or the KEGG database. Category 3 was further divided into: 'c3_promoter_known' and 'c3_promoter_unknown' - genes sharing a motif in the promoter regions (covering -2 kb to 2 kb around transcription start sites) and the motif matches a known transcription factor binding site or the motif does not match any known transcription factor binding site; 'c3_miRNA' - genes sharing a miRNA binding site. Category 4 was divided into: 'c4_bp' - genes sharing biological process ontology terms; 'c4_cc' - genes sharing cellular component ontology terms; 'c4_mf' - genes sharing molecular function ontology terms.

## Abbreviations

CLIP: crosslinking immunoprecipitation; EE: exon-exon; EG: exon-gene; FDR: false discovery rate; GG: gene-gene; GO: Gene Ontology; miRNA: microRNA; NI: normalized intensity; pCastNet: partial Correlation analysis of splicing transcriptome Network.

## Authors' contributions

LC designed the study, developed the method, and performed the analysis. SZ designed and performed the validation experiments. LC and SZ wrote the paper.

## Additional data files

The following additional data files are available with the online version of this paper. Additional data file 1 is a figure showing that the gene-level normalized exon intensity is not stable when the noise level is high. Additional data file 2 is a diagram showing the possible regulation relationships for EG and EE links. Additional data file 3 is a figure showing Venn diagrams of gene pairs with GG, EG, or EE associations. Additional data file 4 is figure showing that exons with EE links and sharing miRNA binding motifs tend to be enriched at the 3' termini of the genes. Additional data file 5 is a figure showing the expression level of FOX-1 and exons with EG links to FOX-1. Additional data file 6 is a proof to show that r_ab·cd _= r_ab·dc _theoretically.

## Supplementary Material

Additional data file 1 Considering a constitutive exon and the gene it belongs to, we simulated exon-level and gene-level intensities according to a bivariate normal distribution. The mean of expression levels is 500. The correlation between the exon-level intensity and the gene-level intensity was set as 0.9 to satisfy that the exon is a constitutive exon. The variance of expression level is 1 (upper panel), 100 (middle panel), and 150 (lower panel). A histogram of gene-level normalized exon intensity from 1,000 simulations is shown. Click here for file

Additional data file 2 Possible regulation relationships for **(a, b) **EG and **(c, d) **EE links. Circles, triangles, and diamonds represent proteins. Click here for file

Additional data file 3 For a pair of genes, if there is at least one EG or EE link, this gene pair was declared to have an EG or EE association. Note that a gene pair may have more than one EG or EE link. Click here for file

Additional data file 4 For each gene, all of the core exons were sorted according to their genomic coordinates (from 5' to 3'). The relative position of the i-th exon was calculated as (I - 1)/(n - 1) where n is the total number of exons. The relative positions were partitioned into 10 windows. The proportion of exons with relative positions falling in each window was counted for exons with EE links and sharing microRNA binding motifs and other exons and denoted as p_1 _or p_2_, respectively. The y-axis represents the p_1_/p_2 _ratio. The error bars represent the 95% confidence intervals of p_1_/p_2_. Notice that p_1_/p_2 _is higher near the 3' regions. Click here for file

Additional data file 5 For each panel, the upper diagram is the NI for exons with EG links to FOX-1 across 11 tissues. The lower diagram is the gene-level expression of FOX-1. Exons with positive correlations to FOX-1 are shown on the left and exons with negative correlations to FOX-1 are shown on the right. Both the NI and the gene-level intensity were standardized across tissues to have mean 0 and variance 1. Each colored line represents a different probe set. In most cases, an exon has one probe set; in other cases, an exon may have multiple probe sets. For human, 71 probe sets corresponding to 63 exons showed a positive correlation with *A2BP1 *expression. For mouse, 19 probe sets corresponding to 17 exons showed a positive correlation. There was a negative correlation for only one exon (exon ID: 103031, assigned to gene *SPTBN1*) in human and two exons (exon ID: 413530 and 632174, assigned to genes *Atp8b1 *and *Depdc5*) in mouse. Click here for file

Additional data file 6 Proof to show that r_ab·cd _= r_ab·dc _theoretically. Click here for file
