# Supplementary figures and images for: Studying alternative splicing regulatory networks through partial correlation analysis
Source: Genome Biol. 2009 Jan 9;10(1):R3. doi: 10.1186/gb-2009-10-1-r3 (PMC2687791; doi:10.1186/gb-2009-10-1-r3)

Additional file 1

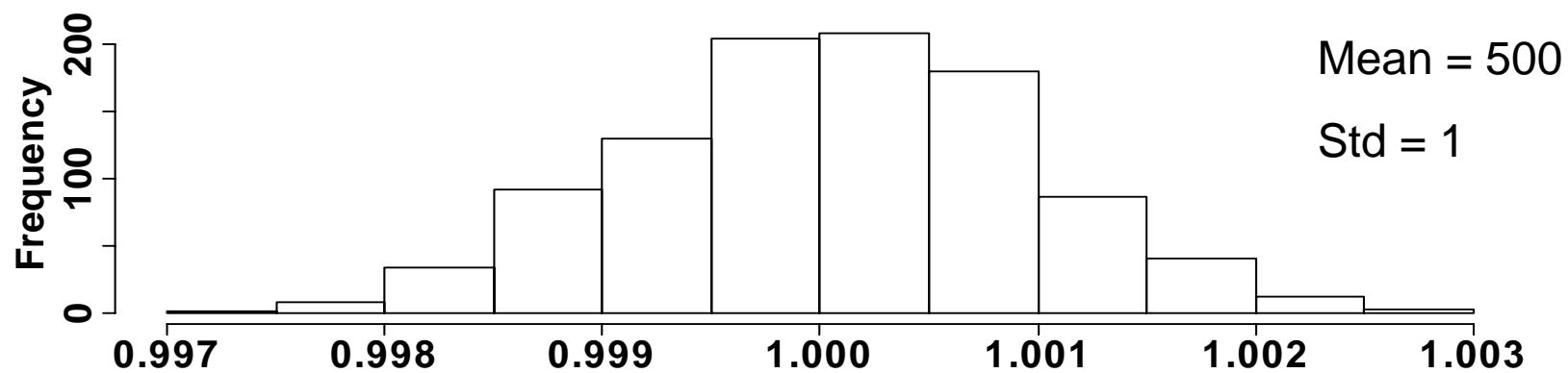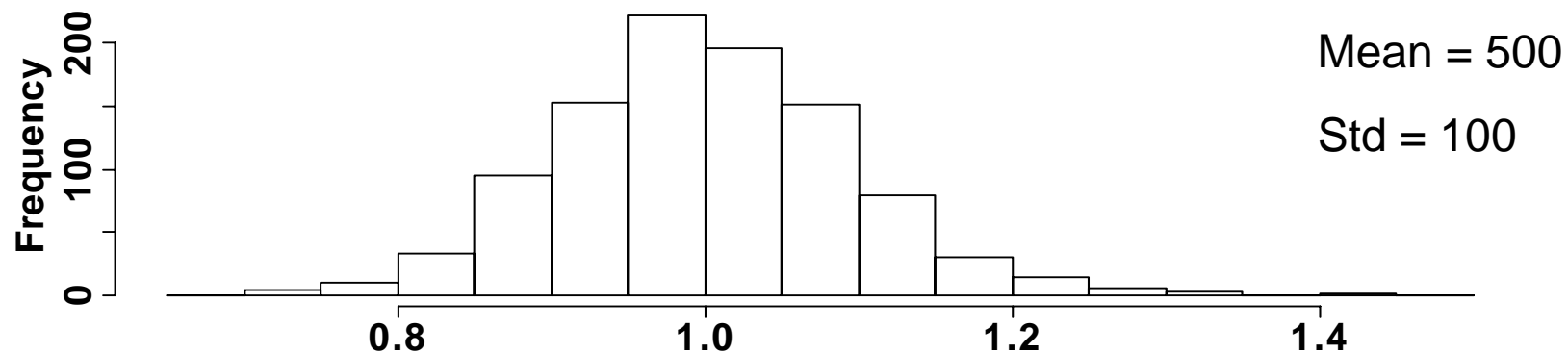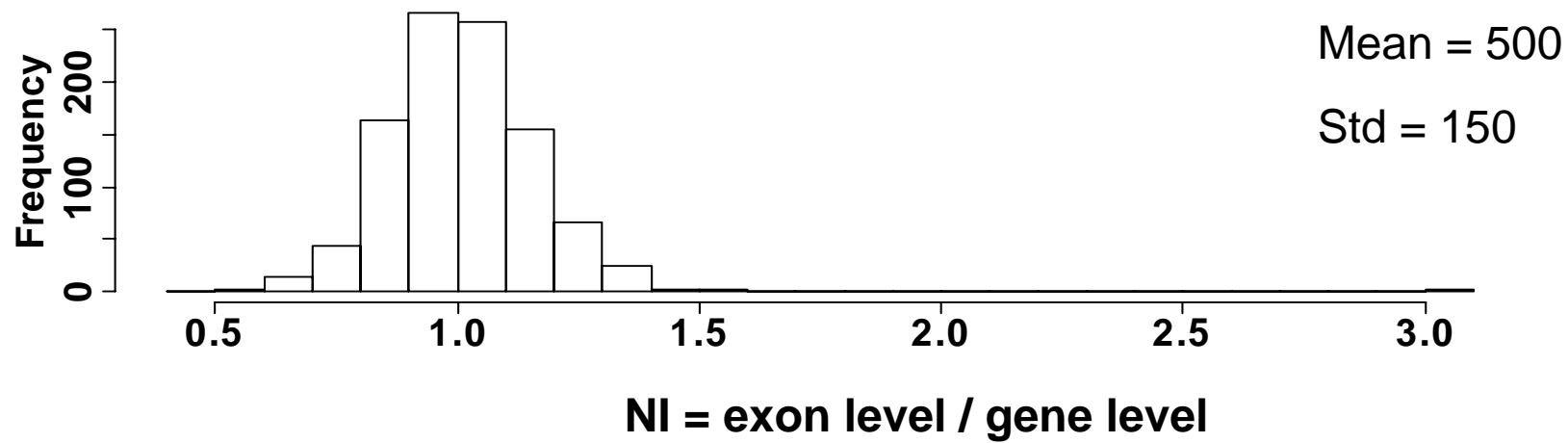

Supplement: Additional data file 1 — Considering a constitutive exon and the gene it belongs to, we simulated exon-level and gene-level intensities according to a bivariate normal distribution. The mean of expression levels is 500. The correlation between the exon-level intensity and the gene-level intensity was set as 0.9 to satisfy that the exon is a constitutive exon. The variance of expression level is 1 (upper panel), 100 (middle panel), and 150 (lower panel). A histogram of gene-level normalized exon intensity from 1,000 simulations is shown. [file gb-2009-10-1-r3-S1.pdf]

## Additional file 2

A

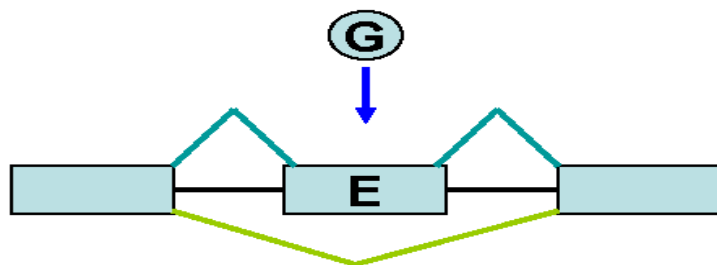

B

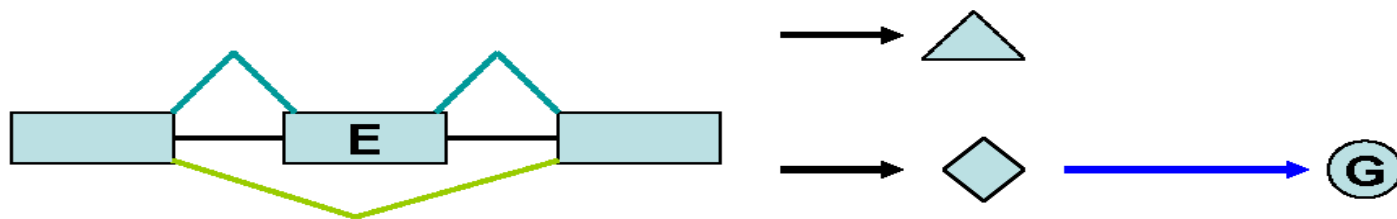

C

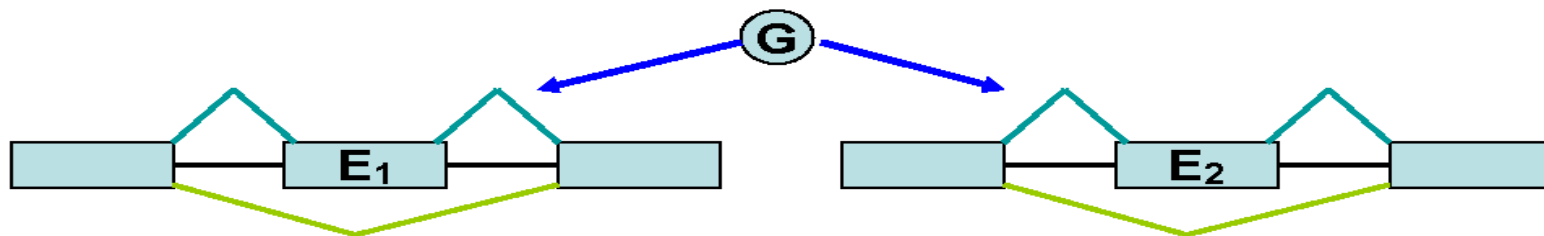

D

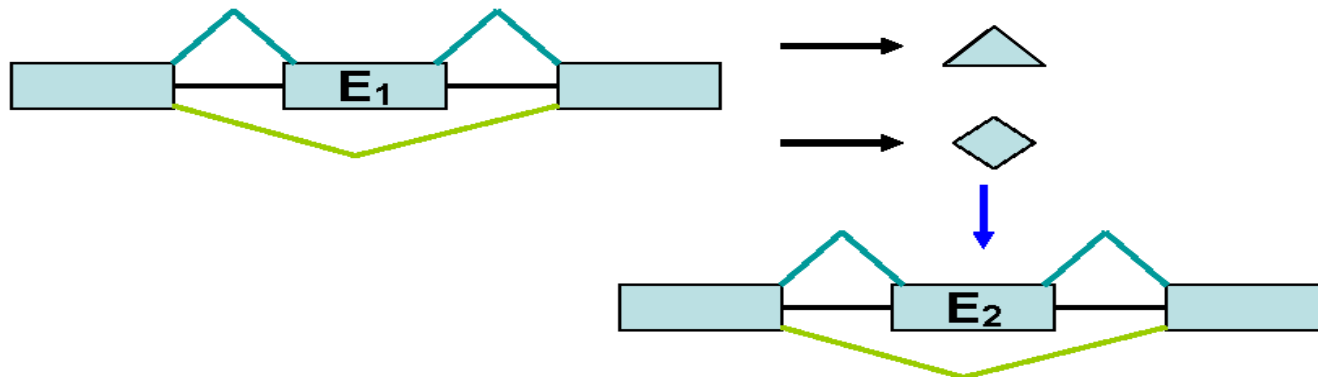

Supplement: Additional data file 2 — Possible regulation relationships for (a, b) EG and (c, d) EE links. Circles, triangles, and diamonds represent proteins. [file gb-2009-10-1-r3-S2.pdf]

**Additional file 3 Human**

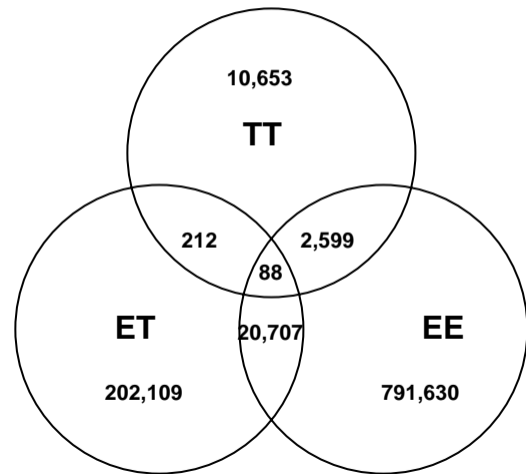

**Mouse**

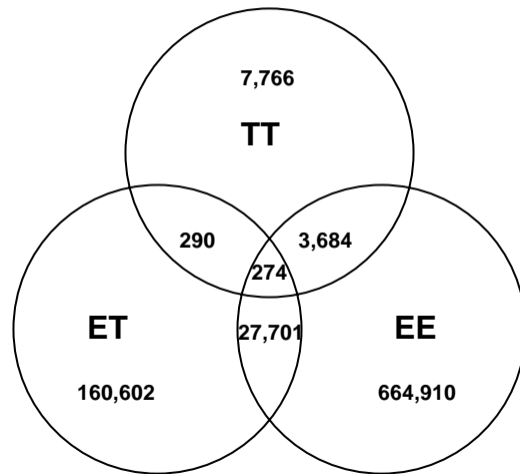

**Rat**

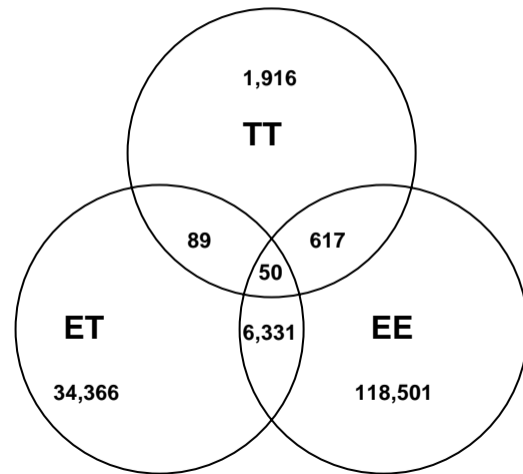

Supplement: Additional data file 3 — For a pair of genes, if there is at least one EG or EE link, this gene pair was declared to have an EG or EE association. Note that a gene pair may have more than one EG or EE link. [file gb-2009-10-1-r3-S3.pdf]

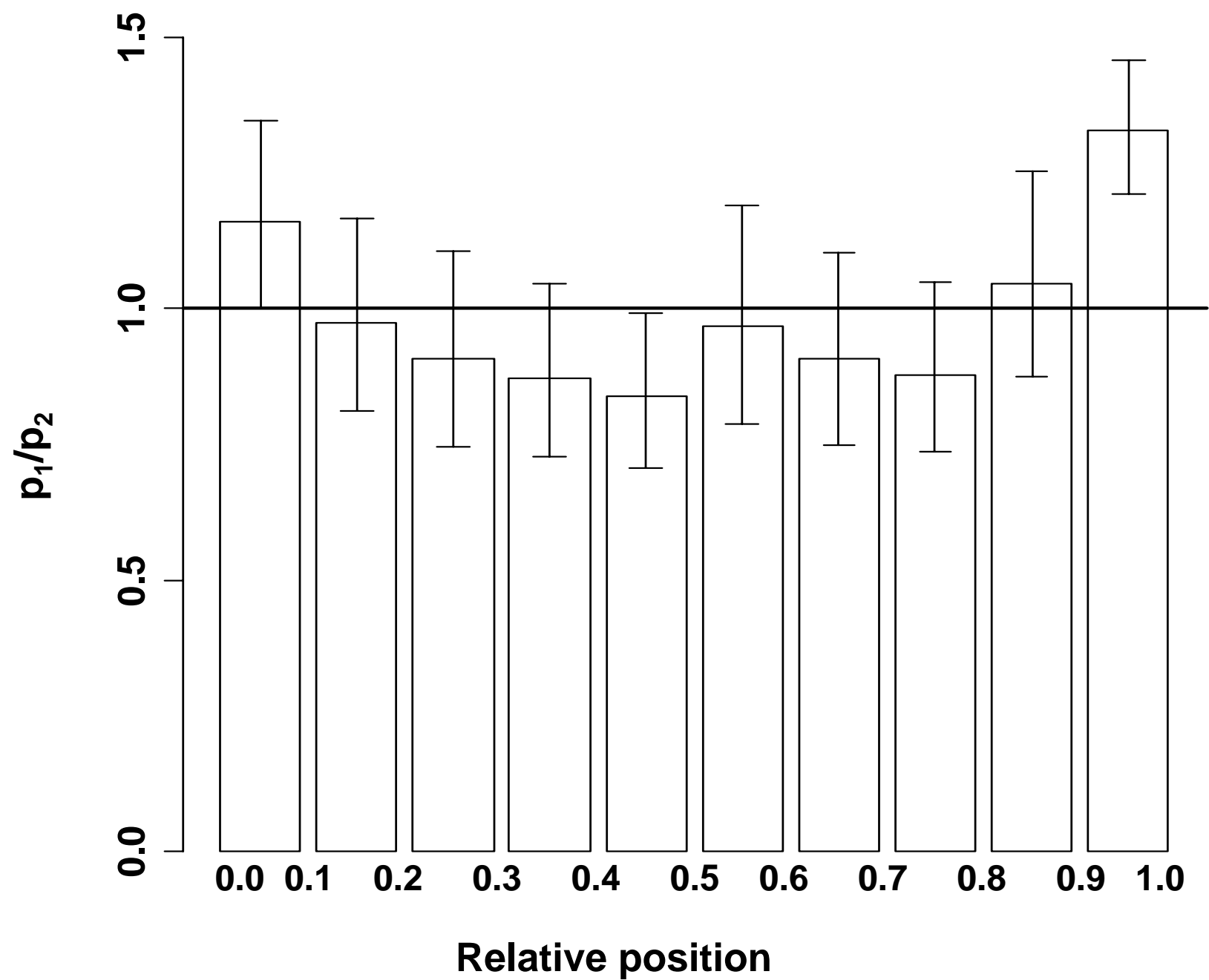

Supplement: Additional data file 4 — For each gene, all of the core exons were sorted according to their genomic coordinates (from 5' to 3'). The relative position of the i-th exon was calculated as (I - 1)/(n - 1) where n is the total number of exons. The relative positions were partitioned into 10 windows. The proportion of exons with relative positions falling in each window was counted for exons with EE links and sharing microRNA binding motifs and other exons and denoted as p1 or p2, respectively. The y-axis represents the p1/p2 ratio. The error bars represent the 95% confidence intervals of p1/p2. Notice that p1/p2 is higher near the 3' regions. [file gb-2009-10-1-r3-S4.pdf]

Human

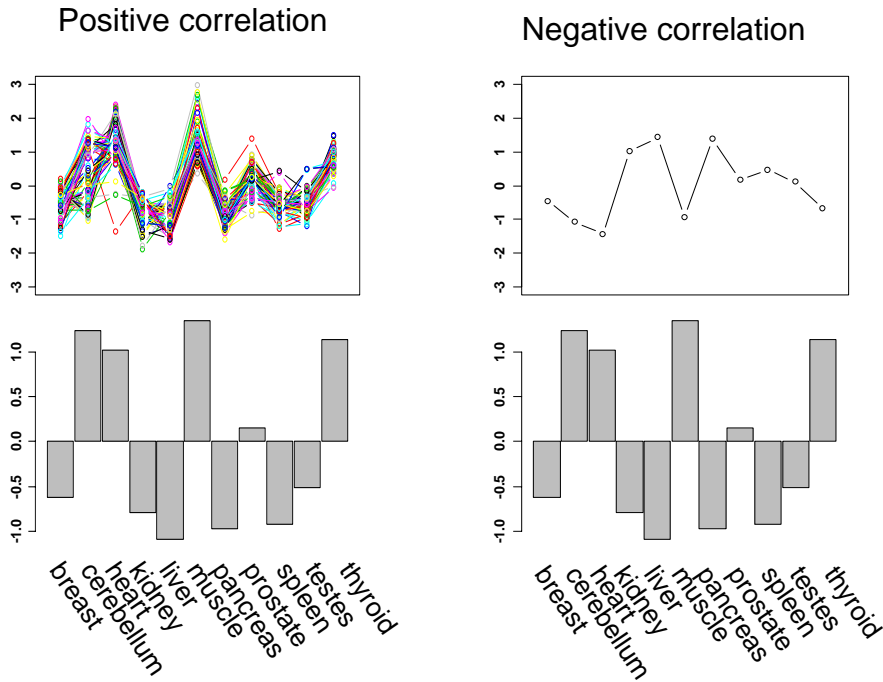

Mouse

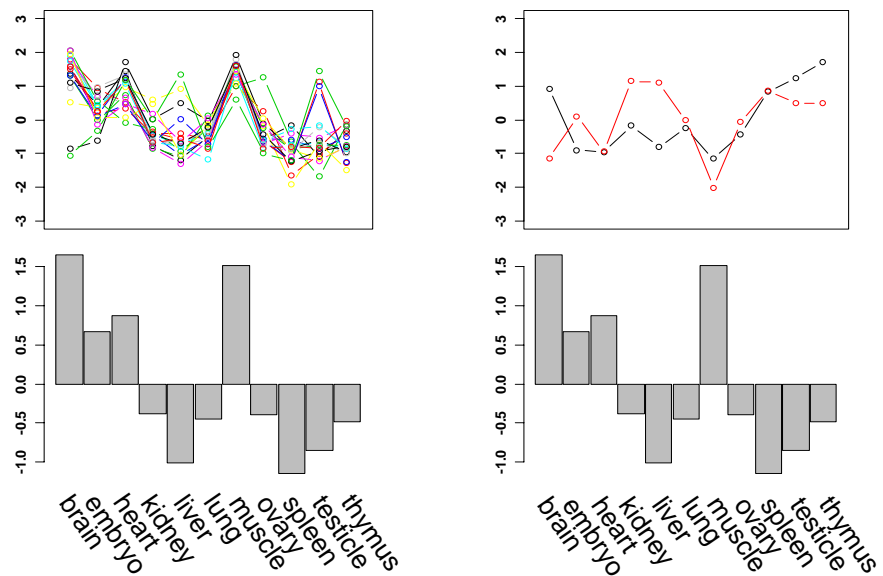

Supplement: Additional data file 5 — For each panel, the upper diagram is the NI for exons with EG links to FOX-1 across 11 tissues. The lower diagram is the gene-level expression of FOX-1. Exons with positive correlations to FOX-1 are shown on the left and exons with negative correlations to FOX-1 are shown on the right. Both the NI and the gene-level intensity were standardized across tissues to have mean 0 and variance 1. Each colored line represents a different probe set. In most cases, an exon has one probe set; in other cases, an exon may have multiple probe sets. For human, 71 probe sets corresponding to 63 exons showed a positive correlation with A2BP1 expression. For mouse, 19 probe sets corresponding to 17 exons showed a positive correlation. There was a negative correlation for only one exon (exon ID: 103031, assigned to gene SPTBN1) in human and two exons (exon ID: 413530 and 632174, assigned to genes Atp8b1 and Depdc5) in mouse. [file gb-2009-10-1-r3-S5.pdf]
