# Supplementary material for: Studying alternative splicing regulatory networks through partial correlation analysis
Source: Genome Biol. 2009 Jan 9;10(1):R3. doi: 10.1186/gb-2009-10-1-r3 (PMC2687791; doi:10.1186/gb-2009-10-1-r3)
Supplement: Additional data file 6 — Proof to show that rab·cd = rab·dc theoretically. [file gb-2009-10-1-r3-S6.pdf]

For variable a, b, c, and d, the pair-wise correlations are:

$$r_{ab} = r_1, r_{ac} = r_2, r_{ad} = r_3, r_{bc} = r_4, r_{bd} = r_5, r_{cd} = r_6.$$

The first-order partial correlations are:

$$r_{ab\bullet c} = \frac{r_1 - r_2 r_4}{\sqrt{(1-r_2^2)(1-r_4^2)}},\ r_{ad\bullet c} = \frac{r_3 - r_2 r_6}{\sqrt{(1-r_2^2)(1-r_6^2)}},\ r_{bd\bullet c} = \frac{r_5 - r_4 r_6}{\sqrt{(1-r_4^2)(1-r_6^2)}},$$

$$r_{ab\bullet d} = \frac{r_1 - r_3 r_5}{\sqrt{(1-r_3^2)(1-r_5^2)}},\ r_{ac\bullet d} = \frac{r_2 - r_3 r_6}{\sqrt{(1-r_3^2)(1-r_6^2)}},\ r_{bc\bullet d} = \frac{r_4 - r_5 r_6}{\sqrt{(1-r_5^2)(1-r_6^2)}}.$$

The second-order partial correlations are:

$$r_{ab\bullet cd} = \frac{(r_1 - r_2 r_4)(1-r_6^2) - (r_3 - r_2 r_6)(r_5 - r_4 r_6)}{\sqrt{((1-r_2^2)(1-r_6^2) - (r_3 - r_2 r_6)^2)((1-r_4^2)(1-r_6^2) - (r_5 - r_4 r_6)^2)}}$$

$$= \frac{r_1 - r_1 r_6^2 - r_2 r_4 - r_3 r_5 + r_3 r_4 r_6 + r_2 r_5 r_6}{\sqrt{(1 - r_2^2 - r_3^2 - r_6^2 + 2 r_2 r_3 r_6)(1 - r_4^2 - r_5^2 - r_6^2 + 2 r_4 r_5 r_6)}},$$

$$r_{ab\bullet dc} = \frac{(r_1 - r_3 r_5)(1-r_6^2) - (r_2 - r_3 r_6)(r_4 - r_5 r_6)}{\sqrt{((1-r_3^2)(1-r_6^2) - (r_2 - r_3 r_6)^2)((1-r_5^2)(1-r_6^2) - (r_4 - r_5 r_6)^2)}}$$

$$= \frac{r_1 - r_1 r_6^2 - r_3 r_5 - r_2 r_4 + r_2 r_5 r_6 + r_3 r_4 r_6}{\sqrt{(1 - r_3^2 - r_2^2 - r_6^2 + 2 r_3 r_2 r_6)(1 - r_5^2 - r_4^2 - r_6^2 + 2 r_5 r_4 r_6)}}.$$

Therefore $r_{ab\bullet cd} = r_{ab\bullet dc}$.
